# Identification of T-Cell Epitopes Using a Combined In-Silico and Experimental Approach in a Mouse Model for SARS-CoV-2

**DOI:** 10.3390/cimb45100502

**Published:** 2023-09-28

**Authors:** Noam Erez, Hagit Achdout, Yfat Yahalom-Ronen, Shimrit Adutler-Lieber, Liat Bar-On, Erez Bar-Haim, Boaz Politi, Einat B. Vitner, Hadas Tamir, Sharon Melamed, Nir Paran, Tomer Israely

**Affiliations:** 1 Department of Infectious Diseases, Israel Institute for Biological Research, Ness-Ziona 74100, Israel; hagita@iibr.gov.il (H.A.); yfatyr@iibr.gov.il (Y.Y.-R.); shimritl@iibr.gov.il (S.A.-L.); boazp@iibr.gov.il (B.P.); einatv@iibr.gov.il (E.B.V.); hadast@iibr.gov.il (H.T.); sharonm@iibr.gov.il (S.M.); nirp@iibr.gov.il (N.P.); 2 Department of Biochemistry and Molecular Genetics, Israel Institute for Biological Research, Ness-Ziona 74100, Israel; liatb@iibr.gov.il (L.B.-O.); erezb@iibr.gov.il (E.B.-H.)

**Keywords:** immune response, T-cell, T-cell epitope, SARS-CoV-2, mouse

## Abstract

Following viral infection, T-cells are crucial for an effective immune response to intracellular pathogens, including respiratory viruses. During the COVID-19 pandemic, diverse assays were required in pre-clinical trials to evaluate the immune response following vaccination against SARS-CoV-2 and assess the response following exposure to the virus. To assess the nature and potency of the cellular response to infection or vaccination, a reliable and specific activity assay was needed. A cellular activity assay based on the presentation of short peptides (epitopes) allows the identification of T cell epitopes displayed on different alleles of the MHC, shedding light on the strength of the immune response towards antigens and aiding in antigen design for vaccination. In this report, we describe two approaches for scanning T cell epitopes on the surface glycoprotein of the SARS-CoV-2 (spike), which is utilized for attachment and entry and serves as an antigen in many vaccine candidates. We demonstrate that epitope scanning is feasible using peptide libraries or computational scanning combined with a cellular activity assay. Our scans identified four CD8 T cell epitopes, including one novel undescribed epitope. These epitopes enabled us to establish a reliable T-cell response assay, which was examined and used in various experimental mouse models for SARS-CoV-2 infection and vaccination. These approaches could potentially aid in future antigen design for vaccination and establish cellular activity assays against uncharacterized antigens of emerging pathogens.

## 1. Introduction

T-cell activity is crucial in clearing viruses from infected tissues and establishing immunity against infections caused by various viral families [1,2,3]. While the humoral response involving antibodies can be significant during primary viral infections, T-cell responses play important roles in the resolution of viral infection, including modulating disease severity in humans and reducing viral loads [4]. Additionally, virus-specific memory T-cells can persist in the body for many years after exposure [5].

The COVID-19 pandemic, caused by SARS-CoV-2, required the rapid development of effective interventions and vaccines. Consequently, understanding the immune response to SARS-CoV-2 infection and vaccination became critical. To evaluate the immune response to SARS-CoV-2 infection or vaccination, it is essential to use tools that efficiently assess the cellular response to the surface glycoprotein Spike (S), which the virus utilizes to bind to target cells through protein trimers [6]. Therefore, the development of vaccines against SARS-CoV-2 is centered around the expression and presentation of S to the immune system. To evaluate the efficacy and strength of the immune response post-vaccination or after exposure to the virus, effective tools and assays are required.

Various methods are available for characterizing the humoral antibody response identifying the type and titers of binding antibodies in human or animal serum. Additionally, virus or pseudoviruses neutralization assays can determine the potential of immune serum to neutralize SARS-CoV-2 [7]. However, these assays alone cannot gauge the magnitude of the cellular response. To address this limitation, our study aimed to develop an assay to quantify the magnitude of the cellular response in mice following SARS-CoV-2 infection or vaccination with SARS-CoV-2.

T-cell activity can be tested ex-vivo using a peripheral blood sample or tissue from humans or animals. In the case of research animals, a cellular assay can also be performed in-vivo. T-cell activity assays are based on the cellular responses resulting from recognising an epitope—a peptide with a specific sequence of amino acids (AA). While the presentation of epitope utilizes major histocompatibility complexes (MHC) class I and class II, recognition is made by the T-cell receptor (TCR). This recognition by the T-cell leads to cytokine secretion, increased division rate, and specific cytotoxic activity [8]. The presence of an antigen is crucial in these assays, which may involve using whole proteins, protein fragments or peptides for stimulation. The peptide length is a significant consideration, as it affects the affinity of epitopes to their receptors and cellular activity [9].

Cytotoxic cellular activity can be assessed by exposing target cells presenting an epitope to T-cells. Various approaches, using different reporter reagents, allow measurement of killing activity either inside the animal (in-vivo cytotoxicity) or ex-vivo after isolating T-cells from the animal and incubating them with target cells [10]. While helpful in assessing T-cell properties, cytotoxic and proliferation assays are less suitable for epitope screening due to the number of animals, the labor involved, and the time required for such a task. Monitoring cytokine production and secretion provides a simple method to evaluate T-cell activation. Activated T-cells produce various cytokines such as the Tumor Necrosis Factor α (TNFα), Interferon-gamma (IFNγ), Interleukin 2 (IL-2), Interleukin 4 (IL-4), Interleukin 10 (IL-10), Granulocyte-Macrophage Colony-Stimulating Factor (GM-CSF), and Macrophage Inflammatory Protein 1-alpha (MIP1α, also known as CCL-3). Quantifying cytokine-producing cells can be achieved through intracellular staining (ICS) or Enzyme-Linked Immunosorbent Spot (ELISpot) assays. Both methods are based on the same principle, but ELISpot is simpler and more efficient [11,12,13,14].

To assess the cellular response to S protein following SARS-CoV-2 immunization or exposure, we aimed to develop a specific and sensitive assay that accommodates various scenarios and routes of exposure. Therefore, characterizing the antigen used to stimulate T-cells, focusing on identifying dominant epitopes in the amino acids (AA) sequence of S, was a crucial step.

In this report, we describe two different and complementary approaches for identifying epitopes and determining their hierarchy. One method involves scanning a library of overlapping peptides representing the AA sequence of the protein. The second method relies on computational (in-silico) screening to predict the affinity of epitopes to specific MHC-I molecule alleles. The identification of these epitopes allowed us to develop ELISpot and ICS assays to evaluate the cellular immune response to SARS-CoV-2 S protein. This combined approach is relatively simple, cost-effective, and can be implemented in a short period.

## 2. Materials and Methods

### 2.1. Animals

All animals in this study were maintained according to the guidelines and regulations for animal experiments at the Israel Institute for Biological Research (IIBR). All animal experiments were approved by the IIBR Institutional Animal Care and Use Committee (IACUC) (protocol numbers M-02-21, M-30-21). C57BL/6J (JAX 00064) and K18-hACE2 (JAX 034860) were purchased from The Jackson Laboratory (Bar Harbor, ME, USA). For infection, virus stocks were diluted in phosphate-buffered saline (PBS) supplemented with 2% FBS (Sartorius Biological Industries, Beit Haemek Ltd., Israel). Animals were anesthetized by intraperitoneal (i.p.) injection of Ketamin and Xylazine (Ketamine 75 mg/kg, Xylazine 7.5 mg/kg in PBS). They were infected by 20 µL intranasal (i.n.) instillation of 50 pfu (SARS-CoV-2) or 10^4^ pfu (Mouse Adapted SARS-CoV-2). Vaccination with VSV-ΔG-spike was performed under anesthesia by intramuscular (i.m.) injection of the described dose in a volume of 50 μL. Animal experiments involving SARS-CoV-2 infection were conducted in a biosafety level 3 (BSL3) facility.

### 2.2. Cells and Viruses

Vero E6 (ATCC^®^ CRL-1586), Calu-3 (ATCC HTB-55), HEK293T (ATCC CRL-11268) and HEK293-hACE2 (GenScript M00770) cells were maintained in Dulbecco’s Modified Eagle’s Medium (DMEM) supplemented with 10% fetal bovine serum (FBS), MEM non-essential amino acids, 2 nM L-Glutamine, 100 Units/mL Penicillin, 0.1 mg/mL streptomycin and 12.5 Units/mL Nystatin (Sartorius Biological Industries, Beit Haemek Ltd., Israel). Cells were cultured at 37 °C, 5% CO2 and 95% humidity air atmosphere. SARS-CoV-2 (GISAID accession EPI_ISL_406862) was kindly provided by the Bundeswehr Institute of Microbiology (Munich, Germany) and propagated in Vero E6 cells. Mouse Adapted SARS-CoV-2 (MA10 variant, NR-55329) was described previously [15,16], obtained through BEI Resources, NIAID, NIH and propagated in Calu-3 Cells. VSV-ΔG-spike was described previously [7,17]. All viruses were tittered by plaque assay on Vero E6 cells as described previously [18], aliquoted and stored at −80 °C until use.

### 2.3. Reagents

Preparation of stabilized soluble SARS-CoV-2 Spike protein was described elsewhere [19]. Peptide synthesis was ordered from Sigma Aldrich (Rehovot, Israel). Spike protein overlapping–peptide library, made of 316 peptides with a length of 15 amino acids and an overlap of 11 amino acids, was ordered from sb-PEPTIDE (Saint Egrève, France), dissolved in DMSO, and used at a final concentration of 2 μg/mL.

### 2.4. ELISpot Assay

Spleens were harvested and processed using a Gentle MACS (Miltenyi, Bergisch Glabach, North Rhine-Westphalia, Germany) according to the manufacturer protocol, filtered, separated on 1.084 gr/mL Ficoll Paque premium (GE17-5446-02, purchased via Sigma Aldrich, Rehovot, Israel), washed in PBS and resuspended in medium. Detection of IFNγ-secreting cells was performed using a Mouse IFNγ single-Color ELISpot kit (Cellular Technology Limited, Biotec, Bonn, Germany). In short, 4 × 10^5^ splenocytes were plated into 96-well ELISpot plates (provided in the kit) in duplicate and incubated for 24 h at 37 °C in the presence of the designated peptides at a final concentration of 2 μg/mL. Visualization of IFNγ-secreting cells was performed according to the manufacturer’s instructions. Quantification of cytokine-secreting cells was determined with an ImmunoSpot S6 Ultimate reader and analyzed with ImmunoSpot software (https://immunospot.com/biospot-software.html (accessed on 20 August 2023) Cellular Technology Limited, Bonn, Germany). Antigen-free cells supplemented with the medium were used as a negative control.

### 2.5. Flow Cytometry

Spleens were harvested and processed using a Gentle MACS (Miltenyi, Bergisch Glabach, North Rhine-Westphalia, Germany) according to the manufacturer protocol, filtered, separated on 1.084 gr/mL Ficoll Paque premium, washed in PBS, and resuspended in medium. For intracellular cytokine staining (ICS), cells were incubated for 5 h with the designated peptides at a final concentration of 2 μg/mL in the presence of a protein transport inhibitor cocktail (eBioscience 00-4980-93, ThermoFisher, Waltham, MA, USA) as directed. Staining for extracellular markers was done using the antibodies listed below for 20 min at 4 °C, washed and surface stained. The cells were then fixed and permeabilized using a Cytofix/Cytoperm kit (554714, BD Biosciences, Moscow, Russia) according to the manufacturer’s instructions before intracellular staining. Samples were collected on an LSR-Fortessa flow cytometer (BD BioSciences), and data were analyzed using FlowJo software v10 (Tristar, Ashland, OR, USA).

The following mAb clones were used for staining: anti-CD3-APC-Cy7 (145-2C11), anti -CD8-Alexa-Fluor-700 (53–6.7), anti-IFNγ-PE (XMG1.2). Aqua or violet Live/Dead cell stain (ThermoFisher) was used for the exclusion of dead cells. All antibodies were purchased from Thermo Fisher (San Diego, CA, USA).

### 2.6. T-Cell Epitope Prediction

Binding prediction of CD8 T-cell epitopes was performed using the Immune Epitope Database (IEDB) (http://tools.iedb.org/mhci/ (accessed on 15 September 2021)) supported by the National Institute of Allergy and Infectious Diseases (NIAID). We used the prediction method NetMHCpan EL4.1 tool [20] for the screening, which focused on H-2-D_b_ and H-2-K_b_ alleles, characteristic of the C57BL/6 strain and its derivatives

## 3. Results

To establish a cellular activity assay, we initially assessed whether an enzyme-linked immunosorbent spot (ELISpot) assay would be suitable. This assay is known for its sensitivity and reproducibility in reporting the activation of individual cells among many cells in a test well [11]. We immunized C57BL/6J mice with VSV-ΔG-spike by intramuscular injection to do this. Vaccination with this vaccine was described by us previously and demonstrated robust humoral response as depicted by ELISA and neutralization assays [7,17]. After one week, we sacrificed the mice and extracted splenocytes from the vaccinated and control naive groups. ELISpot assay was performed using the stabilized S protein as the antigen for stimulation. Negative control wells without stimulation antigen and positive control wells with a mixture of PMA and ionomycin for cell viability were also included. In wells where we used stabilized S protein as the antigen for stimulation, a non-specific IFNγ secretion response was observed for antigen concentrations ranging from 1 μg/mL to 10 μg/mL (Supplementary Figure S1). This response was observed irrespective of whether the cells were derived from vaccinated or control naive animals. The characteristic spots, indicative of T-cell secretion, were not observed in these wells. The robust, unspecific response possibly originated from other cell types, such as macrophages or dendritic cells, which absorbed the foreign antigen and exhibited increased cytokine secretion.

Since using stabilized S as a stimulating antigen for ELISpot assay was not informative, we sought to identify T-cell epitopes that would be used for stimulation as antigen peptides. In-silico epitope screening of the AA sequence of S was performed using the prediction method NetMHCpan EL4.1 tool [20], for the H-2-D_b_ and H-2-K_b_ alleles which are characteristic of the C57BL/6J strain and its derivatives. The screening was performed for 8 AA long epitopes, and the results produced a list of 2533 peptides arranged according to their expected binding scores to MHC class I molecules. Higher scores indicated stronger expected binding to MHC class I. We selected the top 10 epitopes from this list with binding scores above 0.5 (Table 1). Of note, some of the epitopes identified in the screening were also found to be dominant in the S protein of SARS-CoV, likely due to the high homology between the AA sequences of S proteins of both viruses. Notably, all ten epitopes we selected and the first 22 sequences on the list were epitopes that bind to the H-2-K_b_ allele.

**Figure 1:**
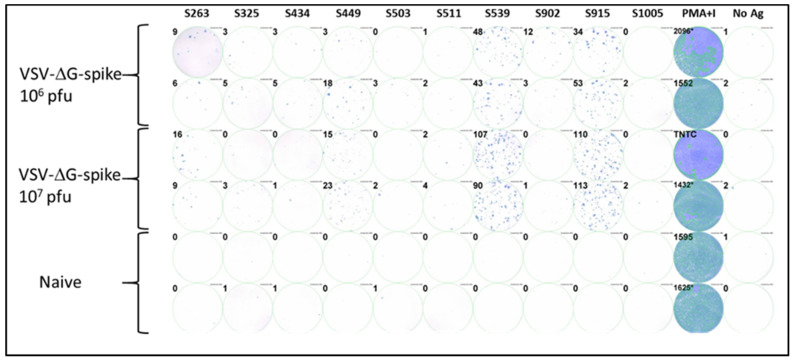
Testing immunodominance of epitopes from in-silico screen following VSV-ΔG-spike vaccination. C57BL/6 mice were vaccinated i.m. with either 10^6^ pfu (n = 3) or 10^7^ pfu (n = 3) VSV-ΔG-spike and sacrificed after seven days. The pool of splenocytes from each vaccinated group was used to evaluate IFNγ secreting T-cells by ELISpot assay in response to each peptide at a concentration of 2 μg/mL. Numbers at the upper left of each well represent the number of counted spots. In the positive control column (PMA + Ionomycin (PMA+I)), asterisk near the numbers represents wells in which some part was saturated; TNTC—too numerous to count.

To investigate the epitopes identified in the computational screen, we synthesized the ten corresponding peptides for further functional biological activity assays. C57BL/6J mice were vaccinated i.m. with VSV-ΔG-spike at doses of 10^6^ pfu or 10^7^ pfu. After seven days, splenocytes were isolated from the spleens of vaccinated and control naive animals, and cellular activity was evaluated using an ELISpot assay with the various synthesized peptides as antigens for stimulation. Positive control with a mixture of PMA and ionomycin was used to assess cellular viability, and wells containing splenocytes from naïve, unvaccinated animals were used as a negative control. Four of the ten peptides tested induced a substantial cellular response, as evaluated by IFNγ secretion in the ELISpot assay. Peptides at positions S539 and S915 elicited strong responses depicted by hundreds of spots observed (Figure 1). Peptides at positions S263 and S449 elicited a weaker but substantial response compared to the negative control. The remaining peptides did not induce a substantial response.

**Figure 2:**
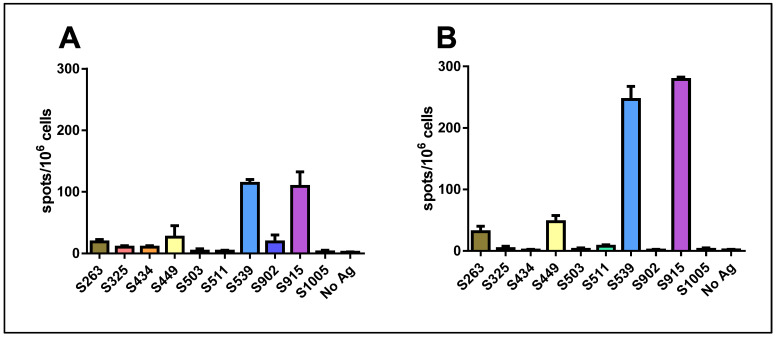
Quantification of T-cell response to different spike epitopes. C57BL/6 mice were vaccinated i.m. with either 10^6^ pfu (**A**) or 10^7^ pfu (**B**) VSV-ΔG-spike and sacrificed after seven days. T-cell response to the different epitopes was evaluated by IFNγ ELISpot assay. For each vaccination dose, groups of three animals were used. Bars indicate means ± SEM. Values are normalized to spots per 10^6^ seeded cells.

When we used a mixture of all ten peptides for stimulation at a concentration of 1 μg/mL for each peptide, a significant response was induced. Surprisingly, this response was not stronger than that obtained with either epitope S539 or S915 alone. Quantification of the results revealed that the hierarchy of epitopes was maintained following vaccination with either 10^6^ pfu (Figure 2A) or 10^7^ pfu (Figure 2B). Yet, an increase in the number of spots was observed following vaccination with the higher dose.

**Figure 3:**
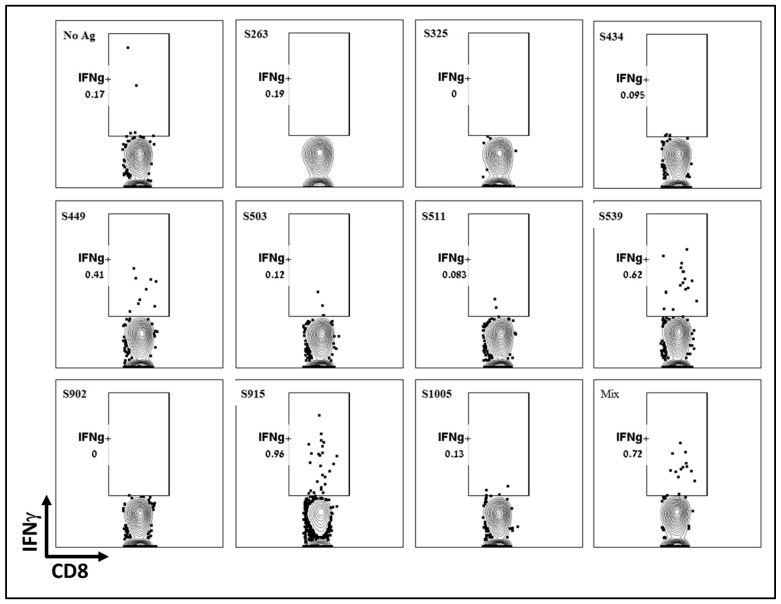
Evaluation of T-cell response to different T-cell epitopes by ICS Following VSV-ΔG-spike vaccination. C57BL/6 mice were vaccinated i.m. with 10^7^ pfu VSV-ΔG-spike and sacrificed after seven days. Splenocytes were used to evaluate IFNγ secreting T-cells by ICS in response to each peptide at a concentration of 2 μg/mL.

To further determine the dominance or hierarchy of the epitopes from the computational screen, we employed intracellular cytokine staining (ICS). Splenocytes from vaccinated and naïve animals were stimulated with the various peptides inducing T-cell-dependent cytokine production. Using a Golgi inhibitor, cytokines accumulated within the cells, and cytokine-positive cells were identified through ICS followed by flow cytometry analysis. Like the ELISpot assay, ICS results showed that the two epitopes, S539 and S915, induced the most robust response, while S449 induced a weaker response (Figure 3). Other epitopes, including S263, which showed a significant response in the ELISpot assay, did not induce a significant cellular response. It appears that, at least for splenocytes, the ICS results did not offer any added value to the ELISpot assay described above. However, it’s worth noting that for technical considerations, ICS may be more informative when performed on cells isolated from other tissues, such as the lungs. It can also detect multiple cytokines in each sample using several antibodies.

**Figure 4:**
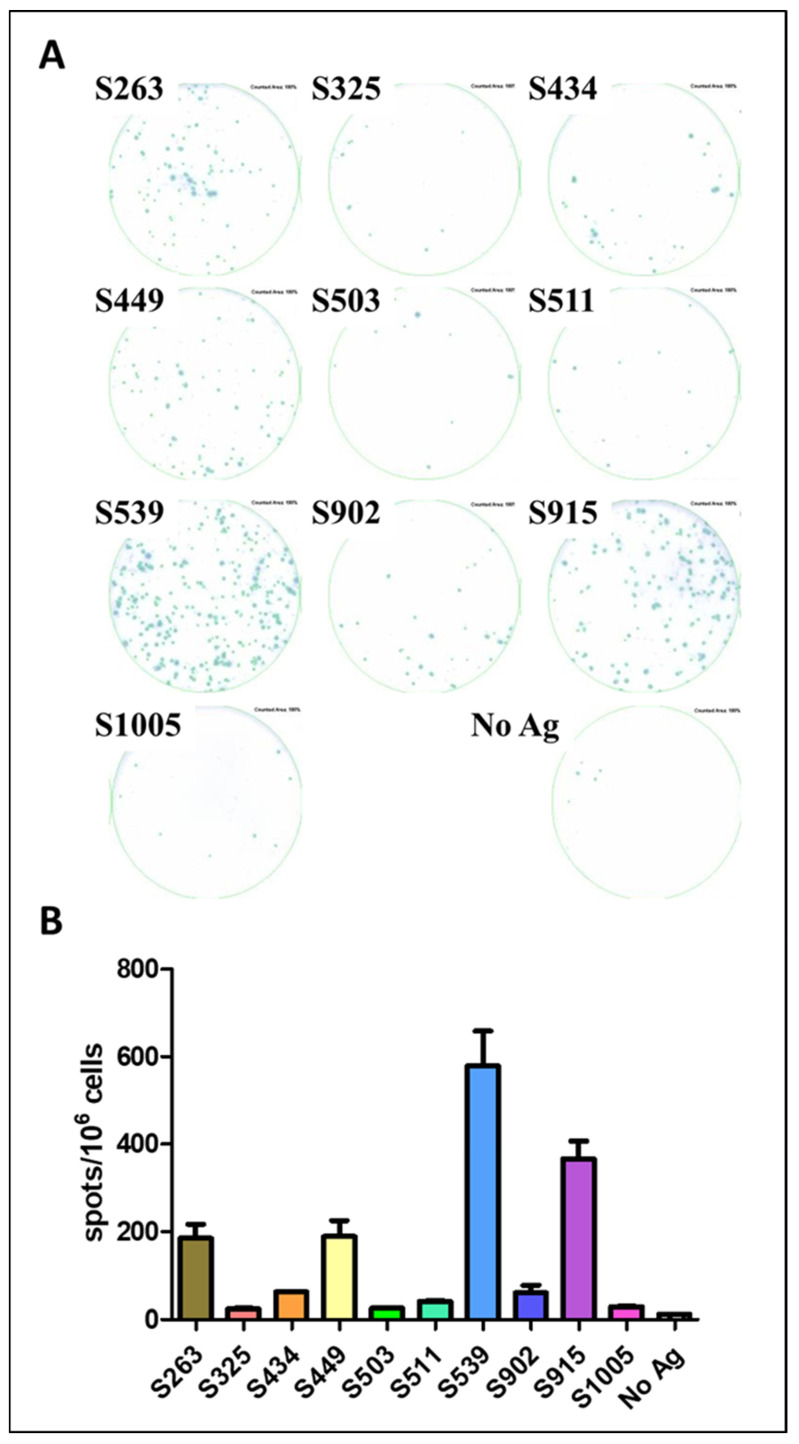
Evaluation of Spike epitopes from in-silico screen following SARS-CoV-2 infection. C57BL/6 mice were infected i.n. with 10^4^ pfu MA10 SARS-CoV-2 and sacrificed after 21 days. T-cell response to different Spike epitopes was evaluated on a pool of splenocytes from infected animals (n = 5) by ELISpot assay (**A**) and quantified (**B**). Bars indicate means ± SEM of 3 replicates. Values were normalized to spots per 10^6^ seeded cells.

The AA sequence in the envelope protein of our VSV-ΔG-spike vaccine virus is based on the sequence of the original SARS-CoV-2 virus [17]. However, the vector used for infection and expression of a viral antigen is an important factor affecting protein expression, processing, and presentation of epitopes [21,22]. To test the potential of the described epitopes to induce T-cell response, we used SARS-CoV-2 infection to determine whether the hierarchy of epitopes is maintained or changed following infection with the pathogen itself. C57BL/6J mice were infected with the MA10 SARS-CoV-2 virus, carrying mutations in the S protein that allow recognition and binding to the murine ACE2 receptor [15]. The infection was carried out at a sublethal dose of 10^4^ pfu, and spleens were removed for ELISpot assay three weeks after infection, using the synthesized peptides as epitopes for stimulation (Figure 4A). The hierarchy of epitopes remained the same as established following vaccination with VSV-ΔG-spike. Epitopes S539 and S915 elicited a strong specific response (Figure 4), approximately 40-fold and 30-fold higher than the background response, respectively, while S263 and S449 induced a specific and significant cellular response, around 15-fold higher than the background response without antigen (Figure 4B).

As mentioned in the introduction, scanning an overlapping peptide library as an antigen for stimulation offers a fast epitope screening method without prior assumptions. This method was described and successfully used in the past to identify SARS-CoV T-cell epitopes [2,23]. MHC molecules can anchor linear peptides longer than the optimal length, leading to T-cell response even if it may be less optimal. Additionally, this library approach can reveal the activation of both CD8 and CD4 T-cells. To assess cellular activity in response to epitopes in the S protein of SARS-CoV-2, we utilized a peptide library containing 316 overlapping peptides. Each peptide was 15 AA long with an 11 AA overlap. C57BL/6 mice, immunized with VSV-ΔG-spike, were sacrificed, and their splenocytes were used for ELISpot activity assay with the different peptides from the peptide library as stimulating antigens. Out of the 316 peptides, only four elicited a strong response above the background: two wells in plate #1 (A10 and B10) and two wells in plate #2 (F7 and G7) (See Supplementary Figure S2). Peptide GWTAGAAAYYVGYLQ and peptide GAAAYYVGYLQPRTF (well A10 and B10, respectively) induced response 3- fold higher than the background of control unstimulated wells. The two peptides that induced the weaker response contained the sequence AAYYVGYL, which corresponds with epitope S263–270. Peptide LVKNKCVNFNFNGLT and peptide KCVNFNFNGLTGTGV (well F7 and G7, respectively) induced a substantial response, approximately 10-fold higher than the background response (Table 2 and Supplementary Figure S2) and contained the immunodominant epitope S539–546. These two epitopes (namely S263 and S539, underlined in Table 2) also emerged in the in-silico epitope screen, and the activation assay derived from it. Peptides containing the AA sequence of S449, which induced a weak response following the computational scan (Figure 1 and Figure 2), did not induce any IFNγ response in the peptide library screen. Furthermore, the immunodominant S915 epitope, which elicited a strong response equal to the S539 peptide (Figure 1 and Figure 2), also did not induce a substantial IFNγ response, as depicted by the peptide library-based ELISpot assay.

**Figure 5:**
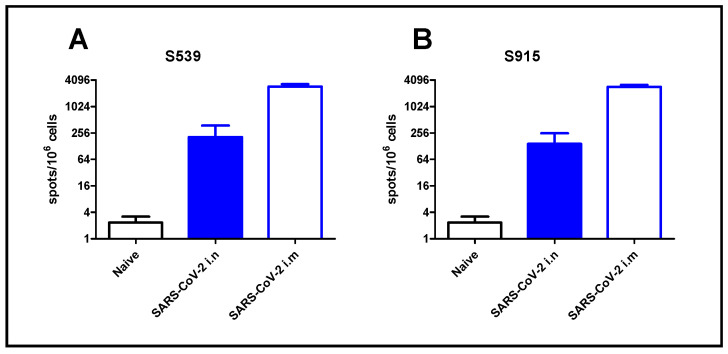
T-cell activation following SARS-CoV-2 infection. K18-hACE2 mice were exposed to SARS-CoV-2 by i.n instillation (2 pfu) or i.m. immunization (10^6^ pfu). T-cell activation was evaluated at day seven by ELISpot assay using peptide S539 (**A**) or S915 (**B**) at a concentration of 2 μg/mL. Bars indicate means ± SEM from 3 animals per group.

To demonstrate the applicability of our approach for epitope screening in the evaluation of the cellular response following SARS-CoV-2 infection, we used the two most dominant epitopes, namely S539 and S915, identified in our screen. For that purpose, we exposed K18-hACE2 mice to a low-dose (2 pfu) of SARS-CoV-2 virus by i.n. instillation, or immunized animals by i.m. injection (10^6^ pfu). Although high, this immunization method was safe, causing only sporadic morbidity [18]. After seven days, the animals were sacrificed, and their spleens were removed and processed. Enumeration of T-cells, specific for the SARS-CoV-2 dominant epitopes, was performed by ELISpot assay using epitopes S539 or S915. Intranasal instillation, even at a very low dose of virus (2 pfu), induced a cellular response, depicted by hundreds of foci per million cells. Immunization by i.m. injection induced a 10-fold increase in cellular response (Figure 5). It should be noted that similar response values were obtained using the S539 epitope (Figure 5A) or the S915 epitope (Figure 5B).

## 4. Discussion

T-cells play a crucial role in the clearance of various viruses, including respiratory viruses such as influenza, Respiratory Syncytial Virus (RSV), SARS-CoV, and MERS [2,24]. Developing assays to evaluate the cellular response following exposure to a pathogen is essential for understanding its immunopathology and developing vaccines against it. Initially, we attempted to develop a cellular activity assay based on ELISpot using the whole stabilized S protein as an antigen for stimulation. However, this approach induced a non-specific reaction with increased IFNγ secretion without characteristic individual spots (Supplementary Figure S1). To overcome this obstacle, we focused on finding specific T-cell epitopes within the S protein.

In this study, we used two different and complementary approaches to screen T-cell epitopes of the SARS-CoV-2 surface glycoprotein (S). One approach involved peptide library screening, which allows a relatively quick and simple screening of many peptides to identify potential epitopes presented by MHC-I and MHC-II molecules. The disadvantages of this method include its relatively high cost and potential availability issues, especially during local or global crises like the COVID-19 pandemic. Additionally, the affinity of MHC-I molecules for peptides depends on their length, with a preference for peptides of 8–9 AAs in the case of mouse MHC-I [25]. The second approach utilized in-silico epitope screening, which predicts the binding strength of a peptide to a specific MHC-I molecule based on its AA sequence and length. This method reduces the number of peptides in the screen and correlates well with in-vitro results [26].

The computational screen identified over 2500 epitopes, with 4 of the top 10 epitopes inducing cellular activity. Among these, epitopes S263, S449, S539, and S915 were identified as dominant (Figure 1). Notably, S263, S449 and S539 were previously demonstrated as epitopes of the S protein of SARS-CoV, while S915 was identified as a unique epitope in this study. The peptide library screening yielded only two regions with a significant reaction in the ELISpot assay, both containing the sequences AAYYVGYL and VNFNFNGL located at positions S263 and S539, respectively. The results from both screening methods were consistent and identified dominant epitopes for CD8 T-cells.

Our in-silico analysis of CD8 T-cell epitopes identified a set of 10 potential immunodominant epitopes, each consisting of 8 amino acids. Subsequent experimental validation using the ELISpot assay confirmed the authenticity of four of these epitopes, with S539 and S915 ranking highest in the hierarchy. Interestingly, an overlapping peptide library screening revealed peptides containing the sequence S539–546 while notably lacking the sequence S915–922. Although we haven’t addressed this discrepancy any further, it is conceivable that including additional flanking amino acids in the library’s peptides (each consisting of 15 amino acids) may have impeded the binding affinity of some peptides but not others.

We also examined the impact of the viral vector used on epitope recognition. In our study, both SARS-CoV-2 and VSV-ΔG-spike expressing the S protein were tested, and the hierarchy of epitopes remained for both vectors. However, the magnitude of the cellular response following SARS-CoV-2 infection was higher than after vaccination with VSV-ΔG-spike, although a lower dose was used for MA10 infection (10^4^ pfu) in comparison to VSV-ΔG-spike (10^7^ pfu). The tropism and superior proliferation ability of the virulent virus may contribute to better activation of the innate immune system and the cellular arm.

Interestingly, a study published previously used an overlapping peptide library for screening of T-cell epitopes in several SARS-CoV-2 proteins [27]. In that study, the authors used Venezuelan Equine Encephalitis replicon particles (VEE VRPs) expressing SARS-CoV-2 viral proteins, including the spike protein, to immunize mice. For epitope scanning, they used an overlapping peptide library to evaluate T-cell response to the different epitopes using ICS for IFNγ. Similar to our study, the authors identified S263 and S538 (similar to S539) as immunodominant epitopes in the spike protein. However, they also identified other epitopes, such as S471, S510 and S820, which were not identified by us.

In contrast, our study identified epitopes S449 (previously identified in SARS-CoV) and S915 (as a novel epitope), which were not described by Zhuang et al. A few differences may explain the results obtained in the two studies. The vectors used for vaccination (VEE VRPs vs. VSV) and the vaccination dose may affect the level of antigen expression and the consequent epitope dominance and hierarchy. Additionally, while in our study, we used a mouse-adapted SARS-CoV-2 virus to directly infect C57BL/6 mice, Zhuang et al. transduced mice by Ad5-ACE2 before infection with SARS-CoV-2.

After identifying the dominant epitopes, we demonstrated the utilization of such epitopes in T-cell activity assays for various SARS-CoV-2 research studies. In the study presented here, we demonstrated the use of two dominant epitopes selected by our screen to evaluate the cellular response following i.n. or i.m. exposure to SARS-CoV-2. This method also served us in additional studies and proved valuable for evaluating the response in different mouse models following vaccination or infection [18,28].

The emergence of SARS-CoV-2 variants of concern (VOC) during the COVID-19 epidemic, raised the troubling notion of compromised immunity in vaccinated populations or in patients that recovered from previous SARS-CoV-2 infection. Few studies have shown that while prior humoral immunity against VOC was affected, the potential cellular immune response remained relatively unaffected by VOC [4,29,30]. In that respect, our selected epitopes remained intact in most VOC; hence, the robustness of cellular assays using these epitopes as stimulating antigens is predicted to remain intact.

In conclusion, using the two described approaches efficiently discovers dominant T-cell epitopes for previously unknown or emerging biological agents. These epitopes can be integrated into various cellular activity assays to evaluate the immune response or develop components against the infectious agent.

## Figures and Tables

**Table 1:** Hierarchy of top 10 predicted CD8 T-cell epitopes.

SARS-CoV-2 Position	SARS-CoV Position	MHC Allele	Sequence	Score
263	250	H-2-K_b_	AAYYVGYL	0.71
325		H-2-K_b_	SIVRFPNI	0.86
434		H-2-K_b_	IAWNSNNL	0.54
449	436	H-2-K_b_	YNYLYRLF	0.73
503		H-2-K_b_	VGYQPYRV	0.71
511	497	H-2-K_b_	VVLSFELL	0.89
539	525	H-2-K_b_	VNFNFNGL	0.97
902	884	H-2-K_b_	MAYRFNGI	0.83
915		H-2-K_b_	VLYENQKL	0.57
1005	987	H-2-K_b_	QTYVTQQL	0.61

**Table 2:** Peptides from library screen which induced T-cell activation.

Peptide Sequence	Spots/Well	Spots/10^6^ Cells
GWTAGAAAYYVGYLQ	59	147
GAAAYYVGYLQPRTF	68	170
LVKNKCVNFNFNGLT	230	575
KCVNFNFNGLTGTGV	125	312
Control—no antigen	8.4 ± 5.9 *	20

* average of 32 wells ± stdev.

## Data Availability

Not applicable.

## Supplementary Materials

The following supporting information can be downloaded at https://www.mdpi.com/article/10.3390/cimb45100502/s1, Figure S1: ELISpot assay using stabilized S; Figure S2: Epitope scanning using SARS-CoV-2 Spike overlapping peptide library.
