# Supplementary material for: Identification of T-Cell Epitopes Using a Combined In-Silico and Experimental Approach in a Mouse Model for SARS-CoV-2
Source: Curr Issues Mol Biol. 2023 Sep 28;45(10):7944–55. doi: 10.3390/cimb45100502 (PMC10605721; doi:10.3390/cimb45100502)
Supplement: Supplementary file 1 [file cimb-45-00502-s001.zip › cimb-2595483-supplementary.pdf]

Supplementary

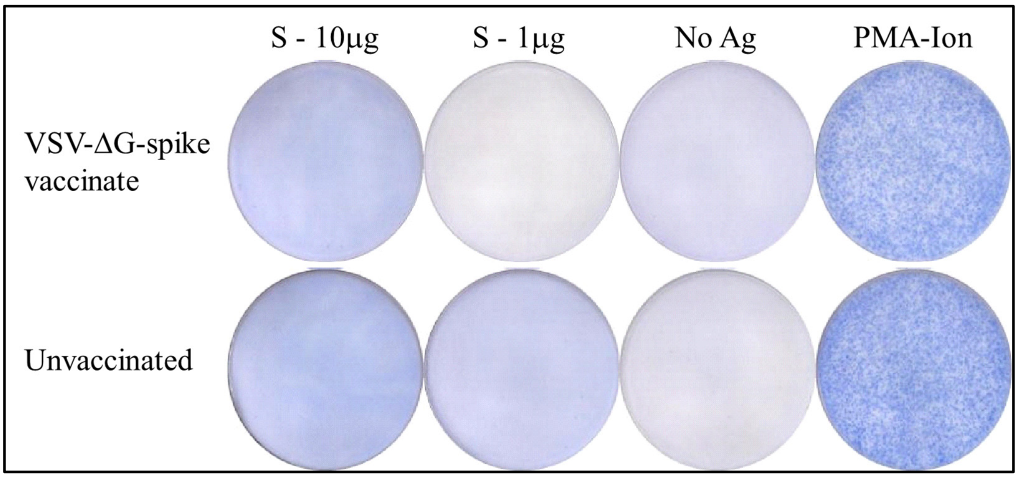

Supplementary Figure S1. ELISpot assay using stabilized S. C57BL/6 mice were vaccinated with VSVΔG-spike by i.m. injection. After seven days, a γ-based ELISpot assay was performed on isolated splenocytes using stabilized S as antigen for stimulation.

plate 1

Spot Counts

	1	2	3	4	5	6	7	8	9	10	11	12
**A**	19	12	11	22	10	21	11	19	16	59	18	21
**B**	10	10	14	14	18	8	9	13	9	68	15	24
**C**	19	12	19	10	3	11	9	5	18	9	13	19
**D**	29	15	10	6	8	10	7	10	4	13	29	13
**E**	16	13	8	10	7	8	4	1	9	24	15	11
**F**	11	24	26	17	20	7	6	18	2	9	15	9
**G**	8	38	15	24	9	16	17	15	8	9	14	13
**H**	10	9	26	22	8	11	13	16	8	14	22	6

Plate 2

Spot Counts

	1	2	3	4	5	6	7	8	9	10	11	12
**A**	9	11	18	13	6	8	3	5	1	13	10	10
**B**	12	4	4	5	2	5	7	16	4	2	9	7
**C**	4	3	8	7	11	4	9	5	7	6	4	6
**D**	4	1	1	3	8	5	7	6	6	5	6	4
**E**	6	10	7	5	1	2	3	7	5	12	5	4
**F**	13	9	5	12	5	7	230	8	9	5	5	8
**G**	3	16	10	7	4	14	125	8	5	2	9	2
**H**	2	4	10	6	4	4	8	7	13	4	4	5

Plate 3

Spot Counts

	1	2	3	4	5	6	7	8	9	10	11	12
**A**	6	11	7	12	10	12	4	15	5	7	7	10
**B**	11	24	6	9	5	8	1	10	18	4	5	14
**C**	8	6	10	8	2	4	5	7	8	12	13	6
**D**	10	11	8	11	7	2	10	7	3	4	13	12
**E**	11	7	5	7	5	6	5	4	3	4	5	5
**F**	4	9	14	2	9	4	7	7	4	6	5	10
**G**	10	11	16	8	12	24	12	14	12	8	3	13
**H**	5	4	3	8	10	18	17	9	6	8	10	3

Plate 4

Spot Counts

	1	2	3	4	5	6	7	8
**A**	7	3	4	2	2	3	7	7
**B**	2	6	4	5	2	1	3	2
**C**	5	7	8	0	10	3	3	2
**D**	0	5	4	5	12	8	4	2
**E**	3	8	7	6	2	2	8	4
**F**	3	6	7	5	1	10	4	4
**G**	4	4	3	4	3	3	2	1
**H**	4	2	0	8	3	2	2	4

Supplementary Figure S2. Epitope scanning using SARS-CoV-2 Spike overlapping peptide library. C57BL/6 mice were vaccinated i.m. with VSVΔG-Spike (10^7pfu). After seven days, splenocytes from vaccinated animals were utilized for IFNγ ELISpot assay using the peptide library as antigens for stimulation. Column 1 of each plate contains negative unstimulated control wells. Highlighted in yellow are wells with a substantial response.
